# Toward better benchmarking: challenge-based methods assessment in cancer genomics

**DOI:** 10.1186/s13059-014-0462-7

**Published:** 2014-09-17

**Authors:** Paul C Boutros, Adam A Margolin, Joshua M Stuart, Andrea Califano, Gustavo Stolovitzky

**Affiliations:** Informatics & Biocomputing Program, Ontario Institute for Cancer Research, University Avenue, Toronto, ON M5G 0A3 Canada; Department of Medical Biophysics, University of Toronto, College Street, Toronto, ON M5G 1L7 Canada; Department of Pharmacology & Toxicology, University of Toronto, King’s College Circle, Toronto, ON M5S 1A8 Canada; Sage Bionetworks, Fairview Ave North, Seattle, WA 98109 USA; Computational Biology Program, Oregon Health & Science University, SW Sam Jackson Park Road, Portland, OR 97239-3098 USA; Department of Biomolecular Engineering, University of California, Santa Cruz, High Street, Santa Cruz, CA 95064 USA; Department of Systems Biology, Biochemistry & Molecular Biophysics, Herbert Irving Comprehensive Cancer Center, Columbia University, St. Nicholas Avenue, New York, NY 10032 USA; IBM Computational Biology Center, TJ Watson Research Center, Kitchawan Road, Yorktown Heights, NY 10598 USA

## Abstract

Rapid technological development has created an urgent need for improved evaluation of algorithms for the analysis of cancer genomics data. We outline how challenge-based assessment may help fill this gap by leveraging crowd-sourcing to distribute effort and reduce bias.

Computational biology comprises three inter-connected activities: algorithm development, validation through benchmarking, and application. In the biomedical sciences, benchmarking occupies a central and indispensable role as it maps algorithms from the space of theoretical possibilities to the realm of practical value. Critically, this process attributes specific probabilities to an algorithm’s discovery of biologically relevant knowledge (measured by the sensitivity of the algorithm) while not overwhelming the researcher with incorrect predictions (quantified by the algorithm specificity). Benchmarking is, however, a complex task, requiring the creation of comprehensive gold standards and the design of sophisticated validation strategies that may require additional experimental data. Indeed, as the use of computational methods in biomedical research becomes widespread, the need for appropriate benchmarking projects, especially those involving community participation, is substantially growing (Table 1). In particular, the rapidly increasing size of whole-genome molecular profile datasets from large sample repositories underscores the importance of benchmarking; it has become virtually impossible to validate algorithmic predictions that are based on such large datasets systematically.

**Table 1:** **Non-comprehensive list of important and current challenge efforts and platforms**

**Challenge**	**Scope**	**Assessment type**	**Organizers**	**Website**
Assemblathon1&2	Sequence assembly	Objective scoring	UC Davis Genome Center	http://assemblathon.org/
CAFA	Protein function prediction	Objective scoring	Community collaboration	http://biofunctionprediction.org/node/8
CAGI	Systems biology	Objective scoring	UC Berkley/University of Maryland	http://genomeinterpretation.org/
CAPRI	Protein docking	Objective scoring	Community collaboration	http://www.ebi.ac.uk/msd-srv/capri/
CASP	Structure prediction	Objective scoring	Community collaboration	http://predictioncenter.org/
ChaLearn	Machine learning	Objective scoring	ChaLearn Organization (non-for profit)	http://www.chalearn.org/
CLARITY	Clinical genome interpretation	Objective scoring and evaluation by judges	Boston Children’s Hospital	http://www.childrenshospital.org/research-and-innovation/research-initiatives/clarity-challenge
DREAM	Network inference and systems biology	Objective scoring	Community collaboration & Sage Bionetworks	https://www.synapse.org/#!Challenges:DREAM
FlowCAP	Flow cytometry analysis	Objective scoring	Community collaboration	http://flowcap.flowsite.org/
IGCG-TCGA DREAM Somatic Mutation Calling	Sequence analysis	Objective evaluation	Community collaboration & Sage Bionetworks	https://www.synapse.org/#!Synapse:syn312572
IMPROVER	Systems biology	Objective evaluation and crowd-verification	Phillip Morris International	https://sbvimprover.com/
Innocentive	Topics in various industries	Objective scoring and evaluation by judges	Commercial platform	http://www.innocentive.com/
Kaggle	Topics in various industries	Objective scoring and evaluation by judges	Commercial platform	http://www.kaggle.com/
RGASP	RNA-seq analyses	Objective scoring	European Bioinformatics Institute	http://www.gencodegenes.org/rgasp/
Sequence Squeeze	Sequence compression	Objective scoring and evaluation by judges	Pistoia Alliance	http://sequencesqueeze.org/
X-Prize	Technology	Evaluation by judges	X-Prize Organization (non-for-profit)	http://www.xprize.org/

The challenges were chosen based on relevance to cancer genomics or the representativeness of a type of challenge. Different challenges specialize in specific areas of research (see ‘Scope’), and may use different assessment types such as objective scoring against a gold standard, evaluation by judges, or community consensus (‘crowd-verification’). Organizers can be researchers from specific institutions (such as universities or hospitals), a group of diverse researchers from academia and industry collaborating in the challenge organization (community collaboration), not-for-profit associations, or commercial platforms that run challenges as their business model (such as Innocentive and Kaggle). Initiatives such as CAFA, CAGI, CAPRI, CASP, ChaLearn, DREAM, FlowCAP and IMPROVER organize several challenges each year, and only the generic project is listed in this table, with the exception of DREAM, for which we also show the IGCG-TCGA DREAM Somatic Mutation Calling Challenge because of its relevance to this paper. More information about these efforts can be found on the listed websites.

Benchmarking is not a matter of simply running a few algorithms on a few datasets and comparing the results. Drawing generalizable conclusions from the exercise requires significant care in design and execution. The maturity of bioinformatics as a discipline has been greatly advanced by the adoption of key principles that guide robust method evaluation, including evaluator objectiveness (lack of bias), clearly defined scoring metrics that align with real-world goals, and the public release of gold-standard datasets and of the results and code of prediction algorithms. Challenge-based (also known as ‘competition-based’) method assessment is an increasingly popular mechanism for benchmarking [1,2]. In this type of study an impartial group of scientists organizes a ‘challenge’ that is based on a carefully curated dataset. This dataset is typically split into a training dataset, a validation dataset (which might be used in real-time leaderboards, typically implemented as a table that reports the comparative performance of the methods under development), and a gold standard (or test) dataset that is withheld from challenge participants and used for final evaluation (Figure 1). Following algorithm development on the training dataset and real-time feedback to participants based on the validation dataset and reported in the leaderboard, the challenge organizers can objectively evaluate the quality of final submitted predictions using a gold-standard dataset. Such a design closely reflects the actual difficulties faced by real-world users trying to determine whether an algorithm generalizes to unseen cases.

**Figure 1:**
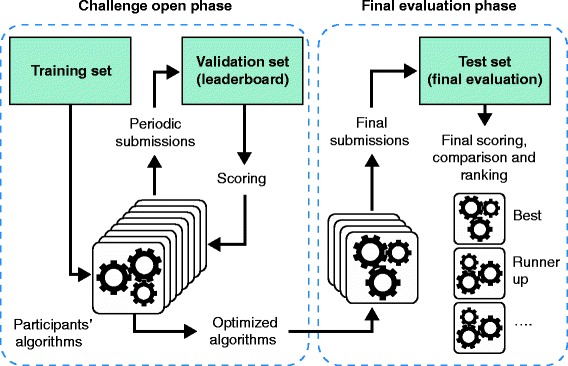
**Typical design of a crowd-sourced challenge.** A dataset is split into a training set, a validation (or leaderboard set) and the test set (or gold standard). Participants have access to the challenge input data and the known answers for just the training set. For the validation and test sets only, the challenge input data are provided but the answers to the challenge questions are withheld. In the challenge open phase, participants optimize their algorithms by making repeated submissions to predict the validation set answers. These submissions are scored and returned to the participants who can use the information to improve their methods. In the final evaluation phase, the optimized algorithms are submitted and evaluated against the final test set (the gold standard), and the resulting scores are used to compute the statistical significance and the ranking of the participating algorithms.

When flawed, benchmarking can lead to the emergence of suboptimal standards that may be applied to many large datasets, imposing an immense cost to the community and creating misleading results. Conversely, the acceptance of knowledge without robust benchmarking can lead to the adoption of inaccurate conventions. For example, during the 1990s, it was generally accepted that the number of loci coding for proteins in the human genome was 100,000, a number that was based on unverified hypotheses [3]. When the human genome was finally sequenced in 2000, the total number of coding loci was found to be a factor of 5 lower. Similarly, a design error in the early implementation of the GC Robust Multi-Array (GCRMA) algorithm, which was revealed by systematic benchmarking of network reconstruction analyses, may have led to the publication of thousands of papers that contain incorrect mRNA abundance profiles before the error was detected and corrected [4]. As a third example, in 2006, a group of Duke University researchers published a pair of high-impact papers claiming accurate prediction of the prognosis of lung cancer patients and of chemotherapy-sensitivity in lung, breast and ovarian cancers. Attempts to reproduce those claims ensued almost immediately, with most of the results falling short of replication because of a combination of programming and data-entry errors, and possible fraud [5]. Proper objective benchmarking by a neutral third-party on private validation data helps to resolve quickly or to detect many of the issues associated with these kinds of studies.

One concern in algorithm benchmarking and validation is that computational biology algorithms are often developed and evaluated by the same researchers. This creates an inherent conflict of interest, where objective assessment of accuracy is polluted by the fact that the developers become simultaneously judge, jury and executioner of the validity of their own work. This can result in biases in study design and over-optimistic performance estimates, whether intentional or unintentional [6]. For instance, the use of non-blinded data in the evaluation by methods developers of their own protein structure prediction methods led, in the early’ 80s, to the false belief that protein structure prediction was essentially a solved problem. Not until 1994, when double-blinded data were used in the first Workshop on the Critical Assessment of Protein Structure Prediction (CASP), was a very different picture revealed [7].

Challenge-based benchmarking efforts, such as CASP [8–10], CAFA [11] and DREAM [12,13], among others (Table 1), offer a robust framework for algorithm evaluation. These efforts have proven the value of engaging both active challenge leaders and motivated algorithm developers to improve their work in a forum that has high visibility and rapid feedback.

We believe that challenge-based methods assessment will play an increasingly important role in standardizing and optimizing the analysis of cancer genomics data, and its broader adoption will drive progress in both algorithm development and biological discovery. Conversely, failing to exploit challenge-benchmarking as a fundamental validation methodology for cancer genomics algorithms may result in lost opportunities to translate results derived from best-in-class methods into patient care.

Here, we provide our perspective on the growing use of challenge-based methods to benchmark algorithms in cancer genomics. We outline the different types of problems faced and some of the key considerations that need to be explored to determine whether a challenge might be successful, and to provide suggestions for challenge organization and execution. Finally, we look to the future and consider how challenge-based assessment may change in the coming decade.

## Challenge design and dynamics

Over the past few years, an established challenge-based design paradigm has emerged in which portions of a private (that is, not globally released) dataset are made publicly available according to a predefined schedule. Such a dataset provides increased user engagement based on continuous feedback; an opportunity for participants to refine and improve their methods on the basis of results obtained throughout the challenge; and multiple independent rounds of validation, which can be used to assess the consistency and robustness of results. After the initial training dataset is made publicly available, a real-time leaderboard can be generated in which the performance of different algorithms is evaluated against a withheld private portion of the data (Figure 1). Previous research has shown that the provision of real-time feedback is among the most important factors in ensuring user engagement in crowd-sourcing projects [14]. (Here, we use the term crowd-sourcing in the sense that a community of tens to hundreds of researchers are engaged in working on the same problem. In other contexts, crowd-sourcing activities may engage different numbers of participants.) After a period of time in which several iterations of the leaderboard can be posted, one of the participating groups is declared the best performer in this initial phase of the challenge, either on the basis of their position on the leaderboard or because they were the first to reach some pre-specified performance level. The challenge may include multiple rounds of assessment based on different portions of the private data. A final round is typically invoked in which methods are rated against a withheld evaluation dataset to determine the overall challenge winner (Figure 1). The most robust validation set is often reserved for this final evaluation - often with larger sample size, newly generated data or prospective validation designed on the basis of challenge results. Each participating team submits a small number (for example, one to five) of independent predictions made by their algorithm(s), which are scored and ranked to determine a winner. Finally, the public release of all of the data kept private throughout the challenge, along with the predictions and ideally source code from each group, provides a long-term resource to spur further development of new and improved methods.

The collection of algorithm source code allows developers to share insights that promote future improvements. If required as part of the final submission, this code can also be used to ensure objective scoring and verification of reproducibility. In the 2012 Sage Bionetworks-DREAM Breast Cancer Prognosis Challenge, participants were required to submit their models as open source R-code [15] that was visible to all participants and executed by an automated system to generate the results reported on the leaderboard. This was enabled by Synapse [16], a software platform that supports scientific challenges as well as large distributed collaborations, such as those in the TCGA Pan-Cancer consortium [17]. Planned challenges, such as the RNA-seq follow-up to the ICGC-TCGA DREAM Somatic Mutation Calling (SMC) Challenge, are considering the use of cloud-computing solutions to provide a central computing facility and a harness upon which contestant code is directly run. This will inherently force the deposition of complete analysis workflows, which can be run routinely on new datasets. Further, this approach would help to standardize application programming interfaces and file formats, such that multiple algorithms use similar inputs and produce easily comparable outputs. This vision of interoperability is shared by many practitioners in the field and has most recently been championed by the Global Alliance for Genomics And Health [18].

Several criteria should be used to help participants limit over-fitting to the training data. Over-fitting is a known peril in statistics, occurring when a predictive model has enough flexibility in its parameters that optimization effectively leads to ‘memorization’ of the training data and an inability to generalize to unseen cases. The most common way to help participants avoid over-fitting, while enabling the testing of their models, is to provide leaderboard scoring that is based on a subset of the private dataset, optimally a subset that is not used in the final evaluation. The latter condition is sometimes not feasible (for example, when the number of patients available to predict clinical outcomes is limited), in which case the leaderboard will be based on data that are also used for the final scoring. If this is the case, limiting the number of submissions can reduce over-fitting.

While most challenges share some common design principles, each research area has its own unique characteristics that require customized experimental designs and consideration of risks and benefits. Indeed, the utility of organizing a challenge to help advance a particular research area depends on a balance between challenge-based benchmarking advantages and limitations, as well as consideration of the potential barriers for participation (Table 2). In the sections below, we highlight three research areas in which rapid development of new algorithms has led to a concomitant need for benchmarking: accurate identification of tumor-specific genomic alterations, association of clinical data with genomic profiles (that is, biomarkers) and identifying network-biology features that underlie cancer phenotypes.

**Table 2:** **Some advantages and limitations of challenge-based methods assessment, along with barriers to participation in them**

**Advantages**	**Limitations**	**Participation barriers**
Reduction of over-fitting	Narrower scope compared to traditional open-ended research	Incentives not strong enough to promote participation
Benchmarking individual methods	Ground truth needed for objective scoring	No funding available to support time spent participating in challenges
Impartial comparison across methods using same datasets	Mostly limited to computational approaches	Fatigue resulting from many ongoing challenges
Fostering collaborative work, including code sharing	Requires data producers to share their data before publication	Time assigned by organizers to solve a difficult challenge question may be too short
Acceleration of research	Sufficient amount of high-quality data needed for meaningful results	Lack of computing capabilities
Enhancing data access and impact	Large number of participants not always available	New data modality or datasets that are too complex or too big poses entry barrier
Determination of problem solvability	Challenge questions may not be solvable with data at hand	Challenge questions not interesting or impactful enough
Tapping the ‘Wisdom of Crowds’	Traditional grant mechanisms not adequate to fund challenge efforts	Cumbersome approvals to acquire sensitive datasets
Objective assessment	Difficulties to distribute datasets with sensitive information	
Standardizes experimental design		

## Analyzing genome assembly and structural variants

**Figure 2:**
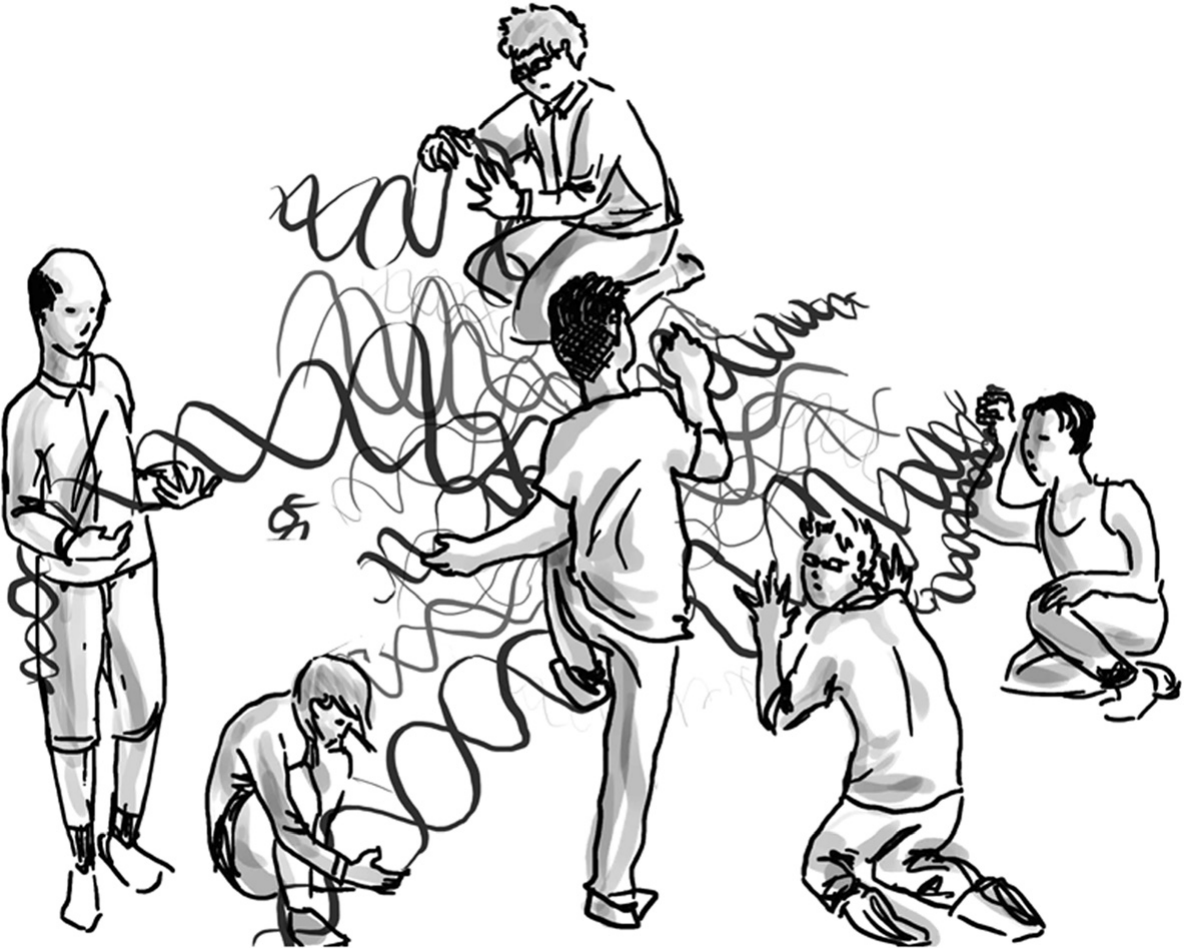
**Different researchers studying the same data may arrive at discordant conclusions.** Benchmarking becomes essential as a way to separate true findings from spurious ones. (Illustration by Natasha Stolovitzky-Brunner© inspired by the parable of the six blind men and the elephant).

Technologies for identifying cancer-related somatic alterations from genomic or transcriptomic data are advancing extremely rapidly. In only 6 years, next-generation sequencing (NGS) has rapidly progressed from the measurement of millions of short sequences (of around 25 bp) to that of hundreds of millions of longer segments (of around 100 bp). This creates an urgent need for on-going benchmarking studies as old algorithms become rapidly out-dated and new algorithmic approaches are required to handle new technologies and new scales of data. Small-scale studies have resulted in dramatic discordance when different researchers apply their algorithms to the same genomic data (Figure 2) [19–21]. These studies have shown that accuracy and generalizability vary dramatically across samples and regions of the genome. The constantly shifting landscape presented by rapidly evolving technologies and tools fuels the urgency in the need to identify the best-performing methods objectively and to re-evaluate them frequently, and to identify particularly error-prone aspects of existing tumor genome analysis methods [22]. Several non-cancer-focused challenge-based benchmarking efforts are on-going, including the Assemblathon benchmarking of *de novo* sequence assembly algorithms [23] and the CLARITY Challenge for standardizing clinical genome sequencing analysis and reporting [24] (Table 1).

Challenge-based benchmarking of methods for somatic variant detection in cancer faces several unique hurdles. First, genomic sequence is inherently identifiable [25], and is thus considered personal health information (PHI) in many countries. This places a burden on challenge contestants to acquire ethics approval from the appropriate authorities, such as dbGaP in the USA or ICGC in Canada. Second, because of the inherent complexity of both the data and file formats, it may be difficult for researchers from other fields to acquire sufficient domain knowledge to compete effectively against domain experts. This point may be ameliorated by gamifying the problem, that is, using game tools that require puzzle solving or geometric thinking to engage users in genomics problems [26,27]. Gamification may not be possible or appropriate, however, because it may require sacrificing domain-specific prior knowledge that is essential to the correct solution. Third, the size of the raw genomic data necessary to perform these challenges creates a ‘big-data’ problem. For example, the ICGC-TCGA DREAM SMC Challenge [28] (Table 1) involved transmitting over 10 TB of data to every contestant, so that each had a copy of the 15 tumor-normal whole-genome pairs. Two different solutions to this problem are to provide access to high-speed, cloud-based download technologies (such as GeneTorrent or Aspera) or to provide co-location of computers and data in a hosted environment [29]. The latter solution has the advantage of providing implementations of the best-performing algorithms in a form that is more readily redistributed to the community, as well as allowing more ‘democratized’ participation for groups that do not have large in-house computing resources. Nevertheless, this solution also has disadvantages: cloud-computing may require additional overhead expenditure for groups that are familiar with developing methods within their local computing environments; many researchers have access to in-house computing options subsidized by their institution and have limited incentive to transfer their analysis to the cloud; and access permissions for some datasets can hinder redistribution through cloud platforms. Furthermore, the assessment of predictions is challenging because the ground-truth for genetic alterations is unknown. The SMC Challenge employs two strategies for evaluation. The first involves an *in silico* method for simulating cancer genomes called BAMSurgeon, which was developed to allow the comparison of methods predictions against a synthetic ground-truth (work by Ewing and colleagues). In the second strategy, targeted deep-sequencing allows prospective validation of a large number of predicted mutations, chosen by an algorithm that most accurately computes false-positive and false-negative rates across submissions. It is unclear how important it is for prospective validation data to be orthogonal to that used by the original challenge participants. Verification in TCGA projects typically relies on deep sequencing using the same technology, but on selected targets and with the construction of new sequencing libraries. This approach assumes that most errors are randomly distributed and/or associated with only a small fraction of reads. The more orthogonal the validation technology, the more this assumption is relaxed. Nevertheless, the error profile of the final evaluation dataset is crucial, and there are currently no error-free approaches to generating this gold-standard data for NGS.

## Finding genomic biomarkers that are associated with phenotype

Once a set of somatic variants have been identified from genomic interrogation of patient-derived samples, one of the most common analyses is to attempt to develop biomarkers that can predict patient survival, response to therapy or other outcomes [30–33]. The development of genomic-based personalized medicine has immense clinical potential, but the optimal approach to predicting such biomarkers *de novo* remains poorly understood and controversial. Indeed, it is widely known that inferred biomarkers are highly sensitive to factors such as choice of algorithm and data pre-processing methods [34–37].

Nevertheless, developing challenges to benchmark biomarker discovery problems is relatively straightforward. Participants are given training data in which features (for example, genome-wide mRNA transcript abundance) are paired with outcome (for example, patient survival) data. Participants are given only the features for the test set and asked to predict the outcome data using a model inferred from the training data. Alternatively, participants may submit trained models as executable code to be run on the test data, thus allowing the test feature data to be hidden from participants [15]. Model results are scored on the basis of the correspondence between predicted and measured outcome data from the test set.

Prediction challenges have been employed in many domains outside of biomedical research [38]. Because biomarker-based challenges fit the setup of the classic supervised machine-learning paradigm, they attract new ideas and participation from the broader machine-learning community. Benchmarking in biomarker discovery is crucial, however, as outlined by the case of the retracted Duke study on chemotherapy selection noted above.

Two key difficulties exist in the creation of benchmarking challenges for biomarker discovery. First, the ideal datasets for biomarker-discovery challenges are uniquely defined, especially when data were collected from large cohorts requiring long-term follow-up or expensive standardized treatment protocols (such as clinical trials). These datasets can potentially lead to high-impact publications or concerns over the intellectual property of the data-generating groups. Second, the potential size of patient cohorts is currently limiting for many biomarker-development questions. If the amount of data available is inadequate, they may not generate enough statistical power to distinguish the performance of the top-ranked groups accurately. These factors also complicate the ability to obtain independent datasets for final method assessment. Despite these problems, several successful challenges pertaining to diagnostics, prognostics and treatment outcomes have been conducted, including the MAQC-II study [39], the IMPROVER Challenge on Diagnostic Signatures [40], the Sage Bionetworks DREAM Breast Cancer Prognostics Challenge [15], and the DREAM AML Treatment Outcome Challenge [41].

## Inferring biological networks underlying cancer phenotypes

Identifying the relationships between biological (transcriptional and signaling) networks and cancer onset and progression is another potential area for challenge benchmarking. Network analysis involves several aspects, including the coherent modeling of different types of alteration and dysregulation events and their integration into a unified network-based model [42–44]. One of the major problems with organizing challenges in this area is that the underlying cellular regulatory networks are mostly unknown, especially in complex systems such mammalian tumor cells. So how can a challenge be organized when a pre-known gold-standard network cannot be defined? Several strategies employed by the DREAM project include using synthetic biology networks [13], *in silico* networks [45], and experimentally assessed bacterial networks [46]. An alternative strategy is to evaluate methods on the basis of their ability to predict the response of a system to a set of perturbations, such as drugs or receptor ligands, as surrogates for predicting the underlying network connectivity [47]. The introduction of ingenious surrogates to the gold standard has enabled the formulation of other network reverse-engineering challenges, such as the 2013 HPN-DREAM Breast Cancer Network Inference Challenge [48]. In this challenge, participants were asked to submit predicted signaling networks that were activated by a set of stimuli in four breast cancer cell lines. These networks were scored on the basis of their ability to identify the set of proteins that are downstream of a given phosphoprotein. The predicted protein set was compared to an experimentally determined set of proteins (the surrogate gold standard), defined as those proteins whose phosphorylation levels were affected by inhibiting that phosphoprotein. Further research on benchmarking network-inference algorithms would be highly beneficial to help advance the field of network biology, whose role in unraveling biological mechanisms in cancer is hard to overestimate.

## The truth is hard to find

From the previous discussion, it is clear that the single most crucial aspect in benchmarking is the definition and assembly of gold standards. A gold standard fundamentally defines the problem under study, and it provides the limiting resolution of error for the overall endeavor. As outlined in this article, gold standards can be defined in several ways. First, a single experiment can be performed with portions of the resulting data used for training and evaluation. This approach avoids experimental inconsistencies, but requires that a large selection of true results is generated prior to the challenge. Simulated datasets are ideal for this strategy but have been criticized as only partially representing a biological system [49]. While validation of simulated data is straight forward, because the ground-truth is completely known, in most cases the value of benchmarking is perceived to be in the ability to assess best-performing methods when applied to real biological data as opposed to simulated data. An important caveat is that the synthetic data may fail to reflect some of the underlying assumptions of the system they attempt to emulate. Indeed, the most common question about simulations is how well they reflect experimental samples [49].

Second, for systems that are difficult to benchmark directly, such as the structure of a biological network, characteristics of the systems can be evaluated instead. These might include the effects of the systems’ perturbation or other phenomena, such as the identification of the networks that best predict patient outcomes.

Third, the results of a study can be validated after the challenge is completed by additional experimental work, either on the same sample or on others. This has the advantage of directly addressing the predictions made by challenge participants, but has the disadvantage of introducing a time lag between challenge completion and the availability of full results. In addition, the effort and cost of follow-up validation may be prohibitive given the resources available to the challenge organizers.

For genomic studies, wet-lab validation can be both time-consuming and expensive. For example, the MAQC study considered approximately 20,000 genes on microarray platforms, but only validated approximately 1,000 (5%) by real-time PCR as a gold standard [50]. Because of this cost, both in terms of time and money, it is critical that a good validation be sufficiently representative, providing similar levels of statistical power for assessing the accuracy of each group. In the context of somatic mutation calling, this means selecting calls that are unique to individual predictors as well as those common to multiple predictors. Indeed, the validation techniques will often be experimentally limited to a subset of results, leaving a bias in the distribution of what is tested. There is thus a clear need for research into the optimal selection of validation candidates in many biological settings. Further, validating a small subset (<10%) of results comes with the possibility, however small, of producing an incorrect relative ordering of different algorithms. In practice, a combination of synthetic and real-world validation is best, and finding the right balance is challenge-dependent.

Finally, some very important elements of cancer genomics are difficult to validate. For example, almost all NGS analyses rely on sequence alignment as a first step. It is, however, very difficult to benchmark the accuracy of an alignment algorithm on real tumor data, because there is no obvious way to create a ground-truth dataset. Thus, rather than benchmarking the aligners, challenges benchmark the results of entire pipelines such as those for detecting somatic variants [28], which may incorporate different aligners and different data pre-processing and statistical approaches. Similarly, it is of great interest to infer cancer-driver genes. Unfortunately, the definition of a ‘driver gene’ (beyond simple statistical recurrence) is unclear, and does not yet allow unambiguous, high-throughput experimental validation. Most experimental techniques in this area probe only one aspect of a driver gene (such as its influence on proliferation or metastasis), while many subtle phenotypes (such as angiogenesis or local spread) are challenging to probe. Also, these designs ignore the potentially polygenic nature of tumor initiation and progression. In designing a new challenge, one of the first questions must be whether or not suitable gold-standard test datasets can be generated.

## Closing considerations

Benchmarking is a fundamental part of computational biology and is increasingly being appreciated by the biomedical community as a whole. Recent benchmarking studies both within [19,51] and outside of cancer genomics [39,52–54] have helped highlight new ways to analyze data and have prompted reconsideration of the error profiles of datasets. Challenge-based assessments have also recently surged in other fields [55] in which the use of incentives (including prizes and prestige) have stimulated increased attention and algorithm development [56].

As the profile of the results of benchmarking studies increases, it is becoming increasingly clear that benchmarking itself is a serious scientific endeavor. The design of a challenge is non-trivial and in some ways is easy ‘to get wrong’ - there needs to be a careful integration between experts in challenge-based benchmarking and domain experts in the challenge topic. At the outset, there is a fundamental requirement for the benchmarking team to foster a community that supports and promotes the exercise. Indeed, some topic areas may be unsuitable to challenge-based benchmarking because a sufficiently big community of interested algorithm developers has not yet emerged (although in these cases, appropriate incentives may be useful in helping to focus attention on a potential challenge topic). Further, the challenge organizing team must be able to assure the broader community of its neutrality and objectivity. There is a clear advantage to building groups of ‘challenge-based benchmarking experts’ who can bring their expertise to diverse topics within cancer genomics, or any other field. Such groups may be well-placed to develop and optimize the statistical methods needed to improve challenge-based benchmarks. Several groups are developing the expertise to facilitate this process, including CASP, DREAM, CAFA and others (Table 1).

Cancer genomics is characterized by rapid technological development, and this trend is likely to persist for many years. As a result, benchmarking cannot be a static endeavor. Rather, each new technology will have its own specific error profiles and distinct algorithms that are used for data analysis. In a world of continual technological and algorithmic innovation, it may be impossible to have definitive, permanent benchmarks, because any effort will be based on a snapshot of technology and will rapidly become out-dated. Instead, a long-running series of ‘living benchmarks’ may allow the co-evolution of benchmarks with technology. In this mutualistic scenario, regular releases of new datasets capturing the current state of experimental methodologies will allow users at any point in time to identify the best tool for their dataset, and algorithm developers to have a dataset suitable for developing and optimizing methods on the latest data.

## Abbreviations

CASP: Critical Assessment of Protein Structure Prediction
GCRMA: GC Robust Multi-Array
PHI: Personal health information
NGS: Next-generation sequencing
SMC: Somatic Mutation Calling
